# Does Coparenting Quality Mediate the Link Between Postpartum Parenting Stress and Perceptions of Emotion Regulation During Parenting?

**DOI:** 10.1111/famp.70084

**Published:** 2025-10-28

**Authors:** Seth D. Finkelstein, Rebecca L. Brock

**Affiliations:** ^1^ Department of Psychology University of Nebraska‐Lincoln Lincoln Nebraska USA

**Keywords:** coparenting quality, emotion regulation, parental self‐efficacy, parenting stress

## Abstract

There is substantial research linking parenting stress to reduced parental self‐efficacy; however, the mechanisms under which this process unfolds are less clear. In a longitudinal, dyadic study of 144 parents and their children spanning postpartum to preschool age, we investigated whether dyadic coparenting quality (i.e., how partners work as a team to parent their children) explains the link between parenting stress and a key domain of parental self‐efficacy—perceptions of emotion regulation during parenting. The study utilized self‐report measures while implementing structural equation modeling (SEM) and actor–partner interdependence modeling (APIM) frameworks. Higher levels of postpartum parenting stress predicted parental perceptions of impaired emotion regulation during parenting interactions with preschoolers through poorer quality coparenting during toddlerhood; however, results suggest that this pathway might be most salient for fathers, and that other unmodeled mechanisms might explain this link for mothers. A partner pathway also emerged such that postpartum parenting stress in one partner undermined parental confidence in regulating emotions during parenting in the other partner by reducing coparenting quality. These findings suggest that coparenting quality may be an essential mechanism driving parental self‐efficacy in the domain of emotion regulation, while undermining the importance of understanding what promotes or hinders parental self‐efficacy, and how this can be implemented into pre‐existing parenting interventions.

## 1 Introduction

General self‐efficacy theories have been applied to several areas in the social sciences (Bandura 1977), and self‐efficacy investigations specific to parenting have become even more prevalent in recent years (Albanese et al. 2019; Fang et al. 2021). Parental self‐efficacy (PSE) is commonly defined as parents' expectations about their parenting abilities (Coleman and Karraker 2003; Vance and Brandon 2017) and has critical implications for parenting quality, parental psychopathology, and child outcomes. For example, higher PSE has been linked with greater positive parenting attitudes (Howard 2006), more responsive parenting (Aranda 2013; Roskam et al. 2016), and higher parental sensitivity (Teti et al. 1996; Wilson et al. 2014). There is substantial research linking lower PSE to parental depression (Albanese et al. 2019), psychological distress in parents (Giallo et al. 2013; Quimby and O'Brien 2006), and lower parental well‐being in fathers (Boyraz and Sayger 2011). Regarding child outcomes, higher PSE has been linked to lower externalizing and internalizing symptoms and better school performance (Ahun et al. 2018; Albanese et al. 2019).

A critical domain of PSE is a parent's ability to regulate difficult emotions that can arise during parenting interactions (Kong and Yasmin 2022). Specifically, adaptive emotion regulation plays a significant role in promoting sensitive and responsive parenting (Crandall et al. 2015), and a lack of adaptive regulation strategies can lead to disjointed and adversarial parenting interactions, resulting in parents feeling less confident in their ability to skillfully parent. Adaptive emotion regulation strategies include problem‐solving, emotional self‐awareness, reappraisal, and acceptance (Aldao and Nolen‐Hoeksema 2010; Aldao et al. 2010; Rodriguez and Shaffer 2021); specifically, emotional self‐awareness (e.g., recognition and understanding of one's emotions) is believed to be a central feature of emotion regulation (Boden and Thompson 2015) with important implications for PSE. Indeed, there is a vast literature demonstrating positive associations between emotional self‐awareness and parental well‐being (e.g., Gratz and Roemer 2004), as well as a decreased risk for parental psychopathology (Mennin et al. 2007). Further, an explicit awareness of emotions plays a critical role in activating other regulation strategies such as reappraisal, which in turn is associated with decreased parental emotional distress (Subic‐Wrana et al. 2014). Thus, the degree to which parents perceive themselves as competent in recognizing and regulating their emotions during parenting interactions is expected to have important implications for building adaptive family dynamics that support healthy child development.

### 1.1 The Role of Parenting Stress in Parental Emotion Regulation and Self‐Efficacy

Parents might feel less capable of regulating their emotions during parenting interactions when experiencing elevated parenting stress, or the “mismatch between perceived resources […] and the actual demands of the parenting role” (Morgan et al. 2005, 61). Crnic and Ross (2017) state that parents can experience a range of stressors related to caregiving responsibilities, children's behavioral/developmental qualities (e.g., promoting the mental and physical well‐being of children), and the parent–child relationship (e.g., feeling overwhelmed when there is disengagement or adversarial dynamics). Parenting stress has been linked to negative parenting (Anthony et al. 2005; Jackson and Choi 2018) and parental psychopathology (Biondic et al. 2019), which is one of the most robust predictors of reduced parenting quality (Taraban and Shaw 2018). Regarding child outcomes, parenting stress has been linked to increased internalizing symptoms in children (Rodriguez 2011) along with externalizing symptoms and behavior problems (Barroso et al. 2018; Morgan et al. 2005).

Parenting stress also has the potential to play a significant role in parents' emotional self‐awareness and perceptions of emotion regulation abilities during parenting interactions. For example, one study found a significant negative association between parenting stress and emotion regulation (Iswinarti et al. 2020), suggesting that increased stress might tax critical regulatory resources needed for coping with stressful parenting interactions. Literature on mindful parenting, the practice of being present within one's emotions as a parent during interactions with children (Kabat‐Zinn and Kabat‐Zinn 1997), also provides an important context for understanding the role of parenting stress in emotional self‐awareness and emotion regulation processes during parenting (Martin and Martin 2021). Bögels et al. (2010) propose a model of mindful parenting whereby reduced parenting stress can increase positive parenting and promote greater emotion regulation during parenting interactions that, in turn, can improve relationships with children.

Meanwhile, parenting stress has been found to be a key pathway through which doubts about PSE develop (Bloomfield and Kendall 2012; Crnic and Ross 2017; Dunning and Giallo 2012). It is important to note that low PSE also has the potential to increase parenting stress (Bloomfield and Kendall 2012; Crnic and Ross 2017); Crnic and Ross (2017) indicate that these constructs likely feed into one another in a cyclic fashion where context and developmental timing are important in determining directionality. To our knowledge, minimal research has examined how elevations in parenting stress subsequently impact various domains of PSE—such as perceptions of emotion regulation abilities—using multi‐wave longitudinal designs.

In sum, research suggests that elevations in parenting stress can undermine critical regulatory resources that parents rely on during parenting interactions, reducing their confidence in their emotion regulation abilities. However, much of this research has been cross‐sectional, and little attention has been paid to the longer‐term implications of chronic levels of parenting stress during the first years of parenting on later parenting experiences and child outcomes. Further, the mechanisms through which parenting stress alters parental perceptions of emotion regulation during parenting remain unclear. Attention to mechanisms within the family system that explain this association might reveal critical modifiable targets for intervention.

### 1.2 Coparenting Quality, Chronic Parenting Stress, and Emotion Regulation During Parenting

Within a family systems perspective (Cox and Paley 2003; Minuchin 1985), consideration of qualities of the interparental relationship (in dual‐parenting households) is important for contextualizing larger developmental outcomes for the entire family unit. The coparenting relationship is defined as “the extent to which partners share leadership and support one another in their mutual roles as architects and heads of the family” (McHale 1995, 985), and dyadic coparenting quality might be one pathway through which parenting stress ultimately impacts parents' perceptions of how well they regulate their emotions when parenting a child.

There is research suggesting that elevations in parenting stress spill over and undermine coparenting effectiveness (Kang et al. 2022). Several studies (Latham et al. 2018; Merrifield and Gamble 2013) link lower coparenting quality to less parental competence and self‐efficacy, whereby coparenting challenges spill over into parenting and undermine PSE. Further, Merrifield and Gamble (2013) found that an undermining coparenting relationship is a robust predictor of low PSE. Negative coparenting dynamics are also related to parental dysregulation, as parents with poorer emotion regulation have more maladaptive communication strategies when interacting with their child, which in turn predicts negative child adjustment (Camisasca et al. 2022). Importantly, interventions targeting healthy coparenting relationships have been developed for couples transitioning to parenthood with promising results (Feinberg and Kan 2008; Solmeyer et al. 2014). Thus, understanding the role of coparenting quality in the link between chronic levels of parenting stress and subsequent perceptions of emotion regulation during parenting may further highlight the need for these interventions and expand their reach to impact other key dynamics in the family system.

### 1.3 The Present Study

The present study identified early developmental pathways leading to parental self‐efficacy in the domain of emotion regulation. Specifically, we investigated the impact of chronic elevations in parenting stress during the first year postpartum on parental perceptions of how well they regulate their emotions during parenting when children were preschool age (5.5 years). Further, we examined whether dyadic coparenting quality during toddlerhood (2 years) was a mechanism through which this effect unfolds. Although research has identified antecedents of general PSE, less is known about the factors contributing to reduced self‐efficacy in *regulating emotions* during parenting interactions, despite the critical importance of being able to set aside difficult emotions when responding to children (Rodriguez and Shaffer 2021). Findings could help identify ways to alter maladaptive pathways anchored in the months following childbirth through which parents lose confidence in their ability to regulate their emotions when parenting (e.g., through interventions targeting early parenting stress and coparenting dysfunction).

We pursued two specific aims. First, we aimed to investigate whether chronic elevations in postpartum parenting stress predict perceptions of impaired emotion regulation during parenting at preschool age for both mothers and fathers. We predicted that greater chronic parenting stress would tax essential regulatory resources and lead to maladaptive thinking about one's effectiveness as a parent in this domain. Second, we aimed to test whether dyadic coparenting quality during toddlerhood mediates the link between postpartum parenting stress and perceptions of emotion regulation during parenting. We predicted that elevations in postpartum parenting stress would spill over into coparenting interactions, undermining the ability of partners in dual‐parenting households to work as a team, leading to a lower‐quality coparenting relationship modeled as a dyadic score (averaging across partner reports). Given the interrelated nature of family subsystems, we predicted that this would, in turn, lead parents to perceive themselves as less capable of regulating their emotions during parenting interactions when their children reached preschool age, consistent with prior research suggesting that coparenting quality impacts PSE (e.g., Latham et al. 2018; Merrifield and Gamble 2013).

## 2 Method

### 2.1 Participants and Procedures

Couples were recruited during pregnancy via flyers and brochures at businesses and healthcare settings frequently visited by pregnant women. Eligibility requirements included: (a) being 19 years of age or older (legal age of adulthood where the research was conducted), (b) English speaking, (c) pregnant at the time of the initial appointment (but not necessarily the first pregnancy to increase generalizability of results), (d) both partners are biological parents of the child, (e) singleton pregnancy, and (f) in a committed intimate relationship and cohabiting. Of the 162 initially eligible couples, three were excluded because of determined ineligibility or invalid data, for a final sample of 159 couples (159 women and 159 men). Due to one mother reporting a miscarriage, one family having a child diagnosed with a developmental disability, and several parents separating, the final sample size for the present study consisted of 144 mixed‐sex couples. Separated families were excluded from the present study to enhance the internal validity of findings, as coparenting can continue after relationship dissolution but does not always (e.g., some separated couples in our sample no longer have contact). This is consistent with Feinberg et al.'s (2012) operationalization of coparenting quality.

Sample demographic characteristics can be found in a supplemental table accessed at https://osf.io/x2avn/. All study procedures were approved by the University of Nebraska‐Lincoln Institutional Review Board. Data were collected when children were aged 1 month (M = 1.12 months, SD = 0.29), 6 months (M = 6.32 months, SD = 0.36), 1 year (M = 12.80 months, SD = 0.76), 2 years (M = 24.50 months, SD = 0.66), and 5.5 years (M = 65.13 months, SD = 0.87). Demographic characteristics were assessed during pregnancy. Data from ages 1 month through 1 year were identified as postpartum, 2 years as toddlerhood, and 5.5 years as preschool age. Parenting stress data were collected from 2016 to 2019, coparenting quality from 2018 to 2020, and perceptions of emotion regulation from 2021 to 2023. Participants were compensated with $25 ($50 per couple) for an at‐home survey at the 1‐month assessment, up to $50 ($100 per couple) for an at‐home survey and semistructured interview via phone for the 6‐month assessment, $100 ($200 per couple) for an in‐person lab appointment at ages 1 and 2 years, and $50 ($100 per couple) for completing an at‐home survey at 5.5 years.

### 2.2 Measures

#### 2.2.1 Postpartum Parenting Stress

Parenting stress was measured via self‐report from both parents when children were ages 1 month, 6 months, and 1 year (data were averaged across timepoints to obtain a robust and reliable measure of postpartum parenting stress). The *Parenting Stress Index* (PSI; Abidin 1995; Dardas and Ahmad 2014) is a 36‐item survey that includes a total score (sum of all 36 items) and three subscales: *Parental Distress* (PD; perceptions of parents' own behavior; e.g., “I feel trapped by my responsibilities as a parent”); *Parent–Child Dysfunctional Interaction* (PCDI; parental expectations of interactions with their children; e.g., “My child rarely does things for me that make me feel good”); and *Difficult Child* (DC; parental perception of child's temperament, demandingness, and compliance; e.g., “My child makes more demands on me than most children”). Each subscale has 12 items with the same Likert scale ranging from 1 (*strongly disagree*) to 5 (*strongly agree*). Subscale scores can range from 12 to 60, and the total score can range from 36 to 180. Internal consistency (Cronbach's alpha) was adequate for the total scale and each subscale at all timepoints: 1 month (Total = 0.96, PD = 0.90, PCDI = 0.92, DC = 0.91), 6 months (Total = 0.95, PD = 0.89, PCDI = 0.93, DC = 0.90), and 1 year (Total = 0.92, PD = 0.87, PCDI = 0.90, DC = 0.86). In the present sample, subscale scores were highly correlated (*r*s ranged from 0.69 to 0.72 for mothers and from 0.62 to 0.77 for fathers), and we focused our analyses on the total parenting stress score. Notably, we observed large correlations among repeated total parenting stress scores (*r*s ranged from 0.65 to 0.75 for mothers and from 0.51 to 0.74 for fathers), and these repeated scores were averaged across timepoints (within partner) to create a robust and reliable score of parenting stress across the first year postpartum. Scores were averaged across timepoints, as opposed to creating a latent variable, to favor parsimony.

#### 2.2.2 Coparenting Quality During Toddlerhood

Coparenting quality was measured when children were 2 years old using the *Coparenting Relationship Scale* (CRS; Feinberg et al. 2012). The CRS is a 35‐item measure of coparenting in two‐parent families. For the present study, a total score was used to capture a shared, general coparenting quality. Example items include “My partner and I have the same goals for our child” and “We often discuss the best way to meet our child's needs.” Participants responded to a 7‐point Likert scale ranging from 0 (*not true of us*) to 6 (*very true of us*) for each item; items were averaged, and total scores could range from 0 to 6. The internal consistency in this sample was adequate (Cronbach's alpha = 0.92). Maternal and paternal reports (*r* = 0.44, *p* < 0.001) were then averaged to create a multi‐informant, *dyadic* score of coparenting quality during toddlerhood (i.e., how well partners come together and navigate coparenting challenges as a couple), as opposed to partialing correlated individual scores by default (Smith et al. 2022). Further, scores were averaged, as opposed to creating a latent variable, to favor parsimony.

#### 2.2.3 Parental Perceptions of Emotion Regulation During Parenting Interactions at Preschool Age

To measure parental perceptions of how well they regulate their emotions during parenting interactions, parents completed the *Regulating Emotions in Parenting Scale* (REPS; Rodriguez and Shaffer 2021) when their children were 5.5 years old (e.g., preschool age). Specifically, the *Adaptive Strategies* subscale was used to capture awareness and modulation of emotions (e.g., “I pay attention to my emotions when I'm with my child,” and “I take care of my emotions before I respond to my child”), including items that emphasize perceived efficacy in managing emotions during parenting interactions (e.g., “I feel like I can handle most problems in the care of my child regardless of how I feel,” and “I can respond to my child's needs despite feeling upset or stressed”). The subscale includes 10 items, uses a 5‐point Likert scale ranging from 1 (*never*) to 5 (*always*), item responses were summed, and scores could range from 10 to 50. Reliability in this sample was adequate (Cronbach's alpha = 0.86). The *Adaptive Strategies* subscale has also demonstrated excellent convergent, divergent, and criterion validity, including strong associations with parenting outcomes (e.g., negative associations with inconsistent discipline and punishment, and a positive association with general positive parenting; Rodriguez and Shaffer 2021). Demonstrating the validity of the REPS in the present sample, *Adaptive Strategies* scores had negative correlations with concurrent child maladaptive emotion regulation measured with the Emotion Regulation Skills Questionnaire (Mirabile 2014; Mirabile and Thompson 2011; *r* = −0.29) and child psychopathology symptoms measured with the Child Behavior Checklist (Achenbach and Rescorla 2000; *r* = −0.40, total problems scale).

### 2.3 Data Analysis

We tested the hypothesized model using structural equation modeling (SEM) with Mplus, version 8.8 (Muthén and Muthén 1998–2017). Data management and analysis procedures for this project were preregistered at https://osf.io/hprk8. For estimating direct paths, we used a robust version of full information maximum likelihood estimation (FIML) to retain all cases despite any missing data and to address non‐normality (Enders 2010). A nonparametric resampling method (bias‐corrected bootstrap) with 10,000 resamples was used to derive the 95% CIs for indirect effects as tests of mediation (Shrout and Bolger 2002). We also implemented key features of actor–partner interdependence modeling (APIM; Cook and Kenny 2005) including (a) actor paths (e.g., maternal parenting stress → maternal perceptions of emotion regulation during parenting), (b) partner paths (e.g., maternal parenting stress → paternal perceptions of emotion regulation during parenting), and (c) correlated residuals of partner outcomes (e.g., maternal parenting stress with paternal parenting stress). We tested for indistinguishability of any two parallel paths across partners (e.g., maternal parenting stress → maternal perceptions of emotion regulation vs. paternal parenting stress → paternal perceptions of emotion regulation) to test for significant gender differences. We tested a constrained model that includes equality constraints in cases of indistinguishable paths; however, we also report results from a model *without* equality constraints to enhance precision in estimating indirect effects separately for mothers and fathers (Sadler et al. 2011).

The model without equality constraints was just identified (saturated) and, therefore, global model fit was perfect. A model implementing equality constraints in cases of indistinguishable paths was overidentified, and we used the following criteria to demonstrate excellent global model fit: CFI > 0.95; SRMR/RMSEA < 0.05 (Brown 2015). We screened for several theoretically meaningful demographic variables for potential inclusion as covariates. Paternal ethnic and racial minority status was significantly correlated with paternal perceptions of emotion regulation during parenting at preschool age (*r* = 0.33, *p* = 0.009), and paternal education level was significantly correlated with dyadic coparenting quality during toddlerhood (*r* = 0.20, *p* = 0.039); thus, both variables were included as covariates in the final model.

## 3 Results

Correlations and descriptive statistics are reported in Table 1. As expected, on average, postpartum parenting stress was relatively low, whereas coparenting quality and perceptions of emotion regulation during parenting were relatively high in this community sample.

**TABLE 1:** Correlations and descriptive statistics.

	1	2	3	4	5
1. Dad REPS (Preschool age)	1.00				
2. Mom REPS (Preschool age)	0.08	1.00			
3. Dad Parenting Stress (Postpartum)	−0.08	−0.07	1.00		
4. Mom Parenting Stress (Postpartum)	0.12	**−0.44** ***	0.14	1.00	
5. Dyadic Coparenting Quality (Toddlerhood)	0.11	**0.28** ***	**−0.17** *	**−0.33** ***	1.00
*n*	60	84	125	132	111
Mean	39.29	38.62	63.17	63.50	5.22
Median	39.00	39.50	60.67	62.17	5.31
SD	4.61	4.98	17.52	16.50	0.50
Min	29.00	20.00	36.00	37.00	3.60
Max	50.00	47.00	128.00	112.00	6.00

*Note:* Significant correlations are bolded.

Abbreviation: REPS, Regulating Emotions in Parenting Scale.

* *p* < 0.050.

*** *p* < 0.001.

Regarding Aim 1, maternal postpartum parenting stress was significantly negatively correlated with maternal (*r* = −0.44, *p* < 0.001), but not paternal (*r* = −0.08, *p* = 0.372), perceptions of emotion regulation during parenting. Partner associations were not significant (e.g., maternal postpartum parenting stress was not associated with paternal perceptions of emotion regulation during parenting at preschool age).

**FIGURE 1:**
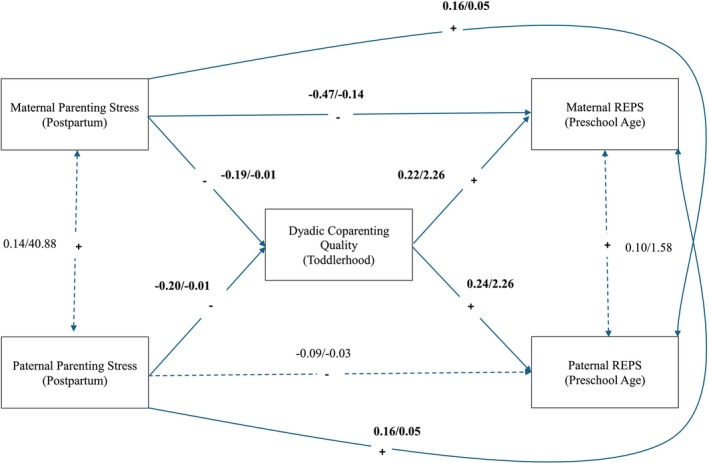
Constrained model of parenting stress, coparenting quality, and perceptions of emotion regulation during parenting. Tests of indistinguishability revealed no significant differences for paths between parenting stress and coparenting quality. *χ* ^2^(1) = 0.135, *p* = 0.714, coparenting quality and REPS, *χ* ^2^(1) = 1.967, *p* = 0.161, and partner paths between parenting stress and REPS, *χ* ^2^(1) = 0.277, *p* = 0.599. Accordingly, equality constraints were implemented for these paths. This model had excellent global fit, *χ* ^2^(3) = 1.974, *p* = 0.578, CFI = 1.00, RMSEA = 0.000, SRMR = 0.036. Standardized/unstandardized coefficients are reported, and solid lines indicate significant effects based on unstandardized estimates (*p* < 0.050). A nonparametric resampling method (bias‐corrected bootstrap) with 10,000 resamples was used to derive 95% CIs for indirect effects. After implementing equality constraints, the overall indirect effect between postpartum parenting stress and perceptions of emotion regulation during parenting at preschool age, through coparenting quality during toddlerhood, was significant regardless of parent gender, 95% CI [−0.029, −0.004]. REPS, Regulating Emotions in Parenting Scale.

**FIGURE 2:**
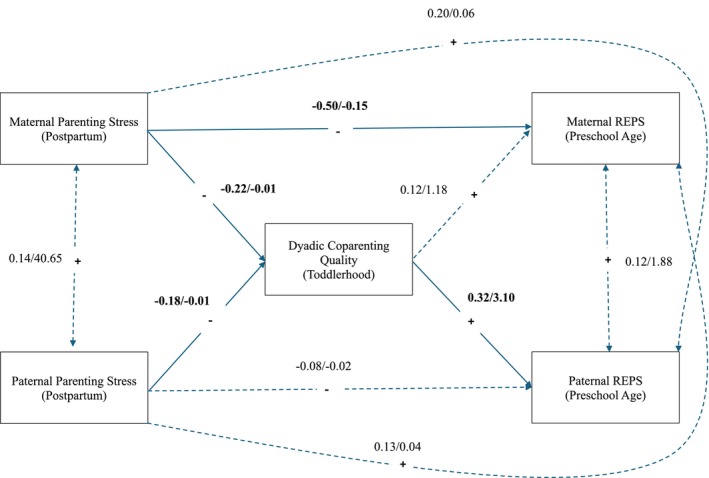
Unconstrained model of parenting stress, coparenting quality, and perceptions of emotion regulation during parenting (allowing gender differences). The unconstrained model, allowing all paths to be freely estimated for more precise estimation of indirect effects for mothers versus fathers, is depicted above. Standardized/unstandardized coefficients are reported, and solid lines indicate significant effects based on unstandardized estimates (*p* < 0.050). A nonparametric resampling method (bias‐corrected bootstrap) with 10,000 resamples was used to derive 95% CIs for indirect effects, where significant effects did not contain 0. When freely estimating parameters, the indirect effect between postpartum parenting stress and perceptions of emotion regulation during parenting at preschool age, through coparenting quality during toddlerhood, was significant for fathers, 95% CI [−0.045, −0.003], but not mothers, 95% CI [−0.033, 0.007]. Further, the indirect partner effect of maternal postpartum parenting stress on paternal perceptions of emotion regulation during parenting at preschool age was significant, 95% CI [−0.049, −0.004], but the indirect partner effect of paternal postpartum parenting stress on maternal perceptions of emotion regulation during parenting at preschool age was not, 95% CI [−0.030, 0.005]. REPS, Regulating Emotions in Parenting Scale.

Results of Aim 2 (Figure 1) suggested that, when controlling for paternal ethnic and racial minority status and education level, parenting stress was significantly negatively associated with perceptions of emotion regulation during parenting for both mothers and fathers through coparenting quality, 95% CI [−0.029, −0.004]; however, when reviewing the results of the unconstrained model (Figure 2), this indirect effect was present for fathers, 95% CI [0.045, −0.003], but not for mothers, 95% CI [−0.033, 0.007]. Further, a significant negative direct effect—independent of coparenting—was observed between parenting stress and perceptions of emotion regulation for mothers, but not for fathers, suggesting this effect might operate through mechanisms other than coparenting for mothers.

### 3.1 Supplemental

Although our hypotheses were focused on dyadic coparenting quality, we explored each partner's unique perceptions of coparenting quality as mechanisms utilizing an unconstrained model. The actor mediation paths were not significant for mothers, 95% CI [−0.061, 0.002], and fathers, 95% CI [−0.071, 0.000]. Further, none of the partner mediation pathways (e.g., maternal parenting stress → paternal perceptions of coparenting → maternal perceptions of emotion regulation during parenting) were significant (i.e., bootstrapped 95% CIs for each indirect effect contained zero).

## 4 Discussion

In a longitudinal investigation of parents and their young children, we sought to investigate whether elevations in chronic postpartum parenting stress predict lower parental self‐efficacy in the domain of emotion regulation (i.e., parental perceptions of emotion regulation during parenting) when children are preschool age, and whether dyadic coparenting quality during toddlerhood is a central mechanism through which this effect unfolds. Consistent with Aim 1 hypotheses, and research suggesting that parenting stress contributes to both decreased emotion regulation and PSE (Albanese et al. 2019; Bloomfield and Kendall 2012; Dunning and Giallo 2012; Iswinarti et al. 2020), we found that greater postpartum parenting stress was associated with lower perceptions of emotion regulation during parenting at preschool age for mothers (e.g., caring for children regardless of whether parents are upset or stressed); however, this was not the case for fathers.

Nonetheless, results of Aim 2 suggest that postpartum parenting stress does ultimately predict self‐efficacy in the domain of emotion regulation for fathers, but through the mechanism of reduced coparenting quality. Notably, although the overall indirect effect was significant in the constrained model (with paths fixed to be equal across mothers and fathers), the unconstrained model suggested this pathway through coparenting is not as salient for mothers and, in fact, there might be other more salient mechanisms through which parenting stress impacts perceptions of emotion regulation during parenting for mothers. Although there is substantial research linking parenting stress to parental self‐efficacy in mothers, there is uncertainty about the specific mechanisms through which this effect unfolds (Crnic and Ross 2017). The development of perceptions of emotion regulation during parenting for mothers might be better explained by internal attributes (e.g., depression), elements of the interparental relationship (e.g., relationship satisfaction), or features of the mother–child relationship (e.g., impaired bonding with infant). Future studies should test alternative mechanisms that might explain the rather robust link between parenting stress and perceptions of emotion regulation during parenting that was observed for mothers in the present study. In contrast, results highlight the potentially salient role of coparenting in *paternal* expectations about their ability to regulate emotions during parenting interactions, which is consistent with past work suggesting that discord in the interparental relationship has a disproportionate impact on fathers with regard to parenting (Cummings et al. 2004).

Notably, the indirect pathway was only significant when modeling a dyadic score of coparenting quality, but not when examining unique perceptions of coparenting quality from each parent. This suggests that parenting stress might perpetuate maladaptive coparenting dynamics at a dyadic level (e.g., less effective management of conflict over childrearing by the couple) and that maladaptive coparenting dynamics—but not unique partner perceptions of the coparenting relationship—have the potential to undermine emotion regulation when parenting.

It was notable that elevations in both maternal and paternal postpartum parenting stress were uniquely and significantly associated with lower dyadic coparenting quality. This is consistent with findings from Kang et al. (2022), who found that greater parenting stress can spill over into the interparental relationship. Indeed, this highlights the indirect role that maternal parenting stress might ultimately play in paternal PSE by undermining effective coparenting. Research has demonstrated that coparenting quality plays an important role in the functioning of the family system more broadly (Feinberg and Kan 2008; Solmeyer et al. 2014). Thus, these results highlight the importance of reducing postpartum parenting stress in both mothers and fathers for bolstering coparenting quality and broader family functioning.

Finally, it was notable that a significant indirect pathway unfolded across partners such that when one parent reported higher levels of postpartum parenting stress, this undermined coparenting quality which, in turn, was associated with lower paternal perceptions of emotion regulation during parenting reported by the other partner. This highlights the interrelated nature of couple dyads and the potential for parenting stress experienced by one parent to spill over into the parenting practices of the other parent through qualities of the interparental relationship.

### 4.1 Limitations, Implications, and Future Directions

Several limitations should be considered when interpreting results of the present study. First, this community sample primarily consisted of White, educated, mixed‐sex parents. Future studies should prioritize replication in more diverse families to enhance generalizability. Additionally, data were solely based on self‐report measures, which may introduce bias and inflate associations due to shared method variance. Future studies would benefit from the incorporation of multi‐method assessments (e.g., observations of coparenting interactions). Further highlighting the utility of observational coding, our approach to averaging partner reports to obtain a dyadic score of coparenting is limited in that it does not account for discrepancies in partner perspectives (e.g., when one partner is relatively satisfied with the coparenting relationship, but the other partner is not). Third, although the *Adaptive Strategies* subscale of the REPS (Rodriguez and Shaffer 2021) captures parental perceptions of how well they can regulate emotions during parenting interactions, this measure was not created to specifically measure parental self‐efficacy; thus, these scores were used as a proxy for parental self‐efficacy in the domain of emotion regulation, in the absence of a direct measure of this construct. Finally, the present study examined longer‐term implications of chronic levels of parenting stress during the first year postpartum on coparenting and parenting into toddler and preschool age; however, parenting stress might fluctuate to a certain degree during the postpartum period, and this fluctuation might drive changes in developing coparenting and parenting dynamics. Future research might investigate the dynamic interplay between parenting stress and coparenting during the postpartum period using multi‐wave designs and growth modeling techniques.

Despite these limitations, results have important research and clinical implications. Although PSE has received increasing attention (Coleman and Karraker 2003; Vance and Brandon 2017), there is a critical need for research investigating the development of specific domains of PSE. That is, what contributes to parents feeling confident in their parenting abilities? What early experiences as a parent undermine one's perceived effectiveness in parenting? Results of the present study suggest that coparenting quality might play a central role in PSE, particularly for fathers, and that elevations in postpartum parenting stress might significantly undermine the quality of the coparenting relationship. Future work is needed to understand how various family dynamics, such as coparenting quality, ultimately bolster PSE across different developmental stages, and the role that gender might play in this process (Cummings et al. 2004). Understanding how these various family dynamics impact outcomes such as PSE can also progress research on parenting stress and its impacts on families (Crnic 2024).

Further, the present study was focused on PSE in the specific domain of emotion regulation. Parents' abilities to regulate their emotions during difficult parenting interactions is a key domain of PSE, and research has connected emotional awareness to aspects of efficacious parenting (Crandall et al. 2015; Subic‐Wrana et al. 2014). Nonetheless, it remains unclear whether parenting stress and coparenting impact PSE in other domains (e.g., being able to physically take care of children or guiding children through specific stages of development), or whether other aspects of the family are more salient mechanisms contributing to the development of other domains of PSE. These questions represent an important future direction to understand how different aspects of parental self‐efficacy are promoted or hindered.

A better understanding of the mechanisms through which PSE develops, uniquely for mothers and fathers and with regard to different features of parenting (e.g., emotion regulation versus physical safety), can inform interventions that protect against negative long‐term outcomes for parents, children, and families (Albanese et al. 2019; Boyraz and Sayger 2011; Giallo et al. 2013; Quimby and O'Brien 2006). Results of the present study identified coparenting as a process promoting paternal perceptions of emotion regulation during parenting, and other research has demonstrated the importance of a high‐quality coparenting relationship for mitigating the negative effects of low PSE on child outcomes (Latham et al. 2018; Merrifield and Gamble 2013). Fortunately, efficacious interventions targeting coparenting already exist (Feinberg and Kan 2008; Solmeyer et al. 2014), and results suggest that early implementation of these interventions (e.g., during pregnancy and postpartum) might play an important role in bolstering PSE and, ultimately, healthy family dynamics and child development (Ahun et al. 2018; Albanese et al. 2019; Aranda 2013; Howard 2006; Roskam et al. 2016; Teti et al. 1996; Wilson et al. 2014). Further, these programs could even be adapted to increase levels of PSE, while being specifically tailored for mothers versus fathers, in tandem with promoting healthy coparenting processes, especially in response to heightened parenting challenges.

## Conflicts of Interest

The authors declare no conflicts of interest.

## Data Availability

The data that support the findings of this study are available from the corresponding author upon reasonable request.
